# Energy evolution and damage ontology modeling of coal destruction at different water contents

**DOI:** 10.1371/journal.pone.0316941

**Published:** 2025-03-06

**Authors:** Yongjiang Yu, Jiaming Liu, Wenjing Guo, Zhiyuan Song, Yuntao Yang, Shangqing Zhao, Dong xu, Zhiqiang Wu

**Affiliations:** College of Mining, Liaoning Technical University, Fuxin, Liaoning, China; Shenyang Jianzhu University, CHINA

## Abstract

The aim of this study was to investigate the energy evolution characteristics and an ontological model of the deformation of coal under different water contents. Uniaxial compression tests were conducted for coal with different water contents, and the analyses were based on the energy principle and the principle of minimum energy dissipation. The results showed that the physical properties of the coal specimens were different under different water contents, the peak strain was positively correlated with water content, and the compressive strength and elastic modulus were negatively correlated with water content. Additionally, the compressive strength and elastic modulus of the coal specimens showed a steep and subsequent slow-change trend. From an energy perspective, the higher the water content of the coal specimens, the higher their energy dissipation at the peak; the smaller the limiting elastic strain energy, the lower the absorbed energy. The principle of minimum energy dissipation was used to deduce the energy evolution and mechanical properties of coal body damage under different water contents, deriving the initial and critical values of damage. The water content of the coal specimens was positively correlated with their initial and critical values of damage, and the relationship with water content was nonlinear. This result was used to establish a stress–strain ontology model for coal rocks with different water contents under uniaxial compression. The model is an improvement over traditional ontology models, addressing the problem of low accuracy in simulations of materials at the compaction stage.

## 1 Introduction

Coal is one of the world’s leading energy sources. The global economy’s rising demand for coal resources and the continuous expansion of the mining scale have been accompanied by a gradual increase in the mining depths and intensity of underground mining [1–3]. During deep coal mining, miners need to consider the effect of groundwater on the coal body when setting aquifer water-proof coal pillars and tunneling, as well as support structures for water-rich roadways. The strength of the coal body is the basis for the coal pillar’s design, and the mechanical properties of the coal body are influenced by its water content. Coal pillars in underground coal mines are approximately in one-way loading states. Therefore, mechanical uniaxial compression tests under different water contents are often conducted on coal bodies, as well as in-depth energy evolution mechanism and damage ontology modeling of the failure. These tests and analyses have important guiding significance for the design of coal pillars and for coal mine safety.

Several scholars have intensively studied the mechanical degradation response and energy damage evolution mechanism of coal with different water contents. Zhang Cun and Ma Jianqi [4] et al. conducted uniaxial compression tests and CT scans on coal with different water contents, analyzing the internal mechanism of water-immersed pore evolution and the weakening of the energy intensity. Yu Yongjiang and Liu Jiaming et al. [5] conducted uniaxial compression tests on coal and sandstone with different water contents; they analyzed the energy dissipation characteristics and damage evolution mechanism of the coal and rock mass at different water contents. Similarly, Xu Qingzhao and Shi Wenbao et al. [6] conducted uniaxial compression tests at different loading rates and performed SEM fracture scanning on coal with different water contents. They determined the mechanical deterioration characteristics and microstructure fracture mechanism of the water-bearing coal samples. Qiu Jilong [7] also performed conventional uniaxial compression tests on water-rich coal and examined the relationship between the water content and mechanical properties of coal. Using the principle of infrared radiation, Ma Kai and Ma Qianqian et al. [8] determined the energy evolution mechanism of the adsorption–desorption process of coal at different water contents. Lu Weiyong and Liu Qi et al. [9] conducted uniaxial compression and acoustic emission tests on coal with different water contents, revealing the macroscopic failure mode of water-rich coal and the change law of the cumulative ringing count. Pan et al. [10] investigated the effect of moisture content on the elastic modulus of coal through uniaxial loading/unloading tests, using coal with different water contents. Wang Kai et al. [11] investigated the effect of moisture on the mechanical properties of raw coal and type coal; they established a segmented damage ontology modeling of coal based on the J. Lemaitre equivalent strain hypothesis, considering water content. Liu Baoxian et al. [12] conducted experiments to study the damage evolution and acoustic emission characteristics of uniaxially compressed coal rocks, establishing a corresponding damage model based on the acoustic emission characteristics. The damage model was used to deduce the damage evolution process of coal rocks. The experiments were conducted using the MTS815 electro-hydraulic servo test system for the rock’s mechanical tests, as well as the 8CHS PCI-2 acoustic emission detection system. Xu Xinwen et al. [13] used the paleomagnetic intensity to determine the characteristics of sedimentary rocks and studied them in detail. Wang Kai et al. [14] conducted experiments under combined static and dynamic loads, and established a staged damage calculation model based on fractal damage mechanics theory to study rock damage. Wang Kang et al. [15] used short-time Fourier transform to analyze the velocity signals in different fracturing areas and studied the changes of rock energy distribution. Evidently, scholars have extensively investigated the mechanical degradation characteristics and damage evolution mechanism of coal under different water contents. However, insufficient attention has been paid to the energy evolution mechanism and damage ontology modeling of the total stress–strain failure of coal.

**Fig 1:**
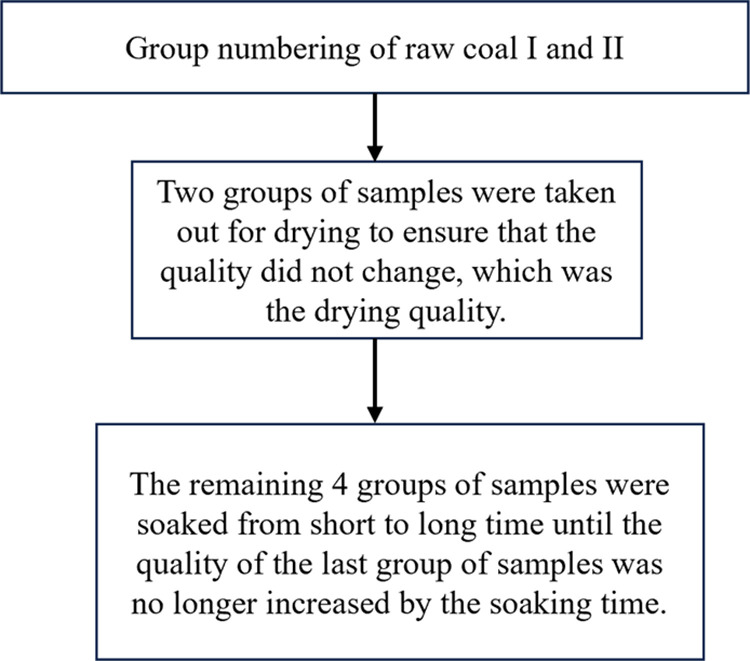
Simple sample preparation flow chart.

Therefore, in this study, a uniaxial compression test of coal with different water contents was conducted to determine the relationship between the water content and mechanical properties of coal. The energy principle was used to analyze the energy evolution mechanism of coal during its deformation and failure under different water contents. On this basis and by using the principle of minimum energy dissipation, the coal damage model was constructed; then, the influence of water content on the damage threshold and critical value was studied. The flow chart of the study is shown in Fig 1. The energy and damage evolution mechanisms of coal under different water contents are the internal mechanism of using rock water injection to prevent rock bursts in underground coal mining. Thus, this study provides a reference value for the prediction and evaluation of water damage in coal rock pillars, the secondary development of FLAC3D and PFC3D numerical simulation software, and the evaluation of rock damage.

## 2 Preparation of specimens and test methods

**Fig 2:**
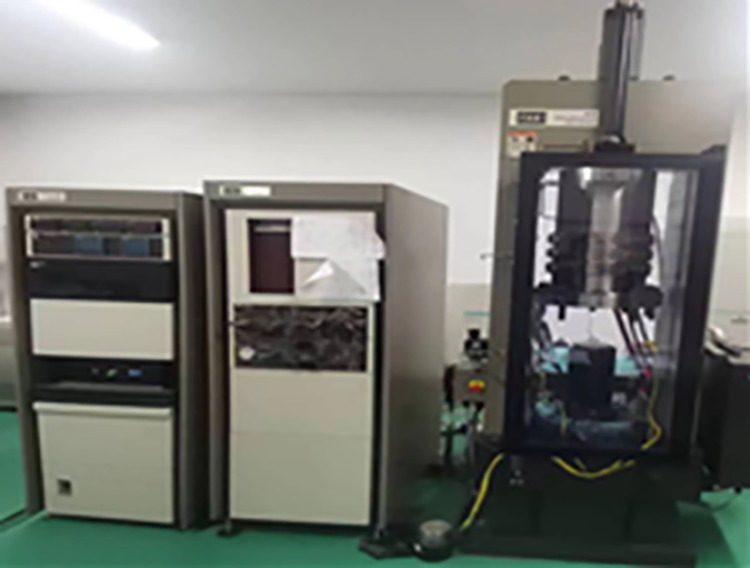
MTS815 fully digital hydraulic servo test system.

**Fig 3:**
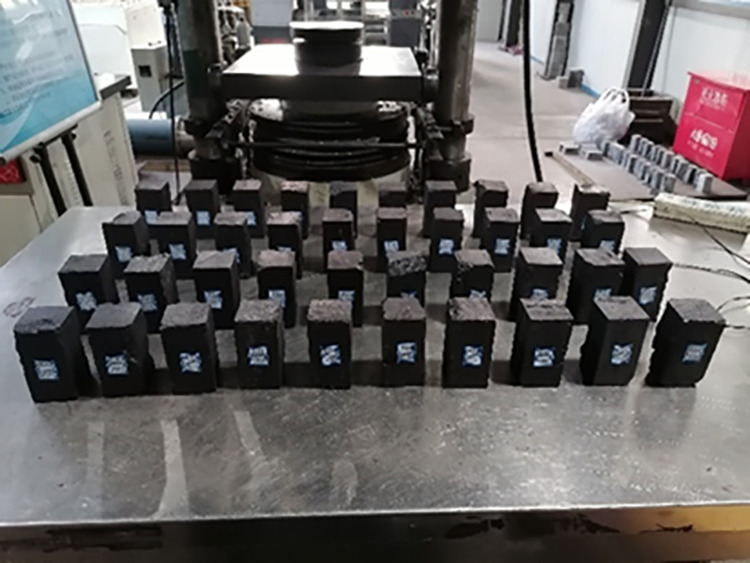
Coal specimens with different water contents.

**Fig 4:**
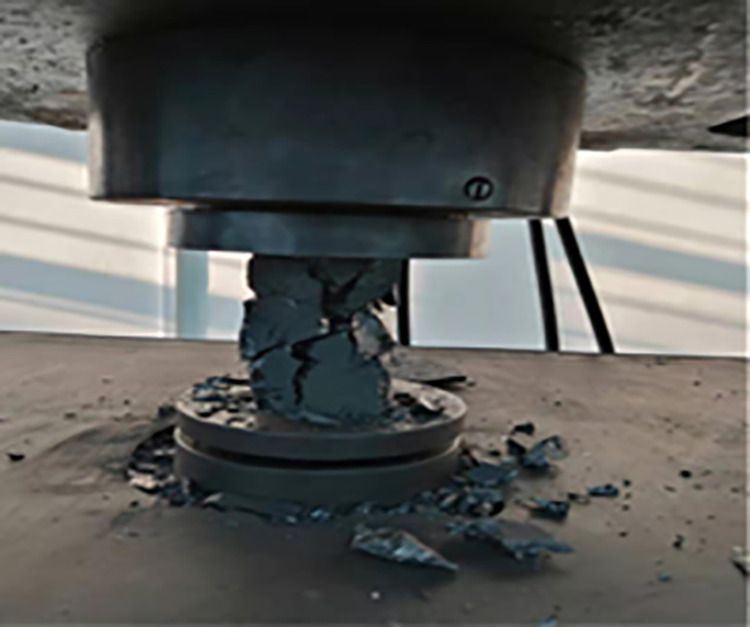
Uniaxial compression test of coal specimen.


The uniaxial compression test of coal was conducted in the State Key Laboratory of Liaoning Technical University. The MTS815 fully digital hydraulic servo-testing system was used, with a load application method based on displacement control. The loading speed was set to 1 × 10^‒3^mm/s, and data were collected at intervals of 1 s. The stress–strain curve of the entire destruction process was obtained. The coal sample was obtained from the 4–1 coal seam of Shuangma No. 1 Mine in Yinchuan, Ningxia. The coal system comprised 50 mm ×  50 mm ×  100 mm column specimens, and the ends of each specimen were polished. There were five water content states of the specimens, and the preparation of coal with different water contents was in accordance with previous research [11]. Two samples were prepared for each water content state, making a total of two groups, which were labeled original coal I and original coal II. Figs 1–4 illustrate the experimental process.

$$\omega_{\text{c}}=\frac{m_{\text{c}}\text{-}m_{\text{d}}}{m_{\text{d}}}\times 100\%$$ (1)

where m_c_ is the mass of the coal in the watery state; m_d_ is the mass of the coal in the dry state; ω_c_ is the water content of the coal.

### 2.1 Results and discussion

**Fig 5:**
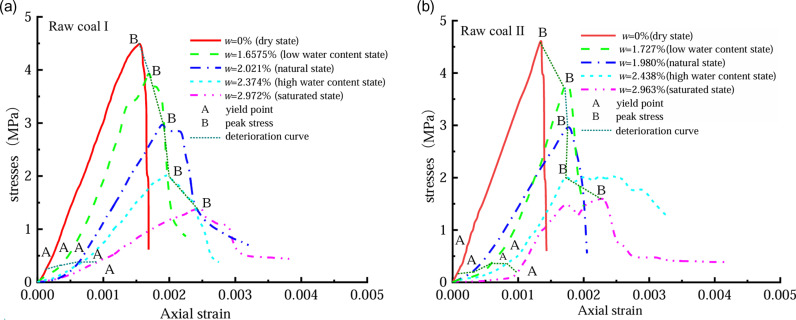
Stress–strain curves of coal rocks with different water contents:(a) Stress–strain curve of raw coal I with different water contents; (b) Stress–strain curves of raw coal II with different water contents.

Fig 5 shows the stress–strain curves of coal samples undergoing uniaxial compression tests at five distinct levels of moisture content. In the dry state, the coal had a shorter duration in the compaction stage, the proportion of linear elasticity before the peak was larger, and the stress after the peak fell rapidly, which was because no moisture filled the pores of the coal with the lubrication of water. Moreover, the tiny coal fissures were relatively difficult to slide against each other, increasing the brittleness of the coal. As the water content increased, the proportion of the compaction stage of the coal increased, the proportion of the linear elasticity stage decreased, and the yield stage became more significant. The stress–strain curve shows an upward-convex shape in the post-peak loading and maintains a large residual strength. This is because the pores of the coal are filled with different amounts of moisture, and the lubricating effect of moisture facilitates the expansion and connection of microscopic cracks and pores inside the coal rock; hence, the coal rock becomes less brittle but more plastic [16–20]. Water content significantly affects the mechanical properties and damage characteristics of coal, as observed in a previous analysis [21–23].

## 3 Mechanical characterization of coal with different water contents

**Fig 6:**
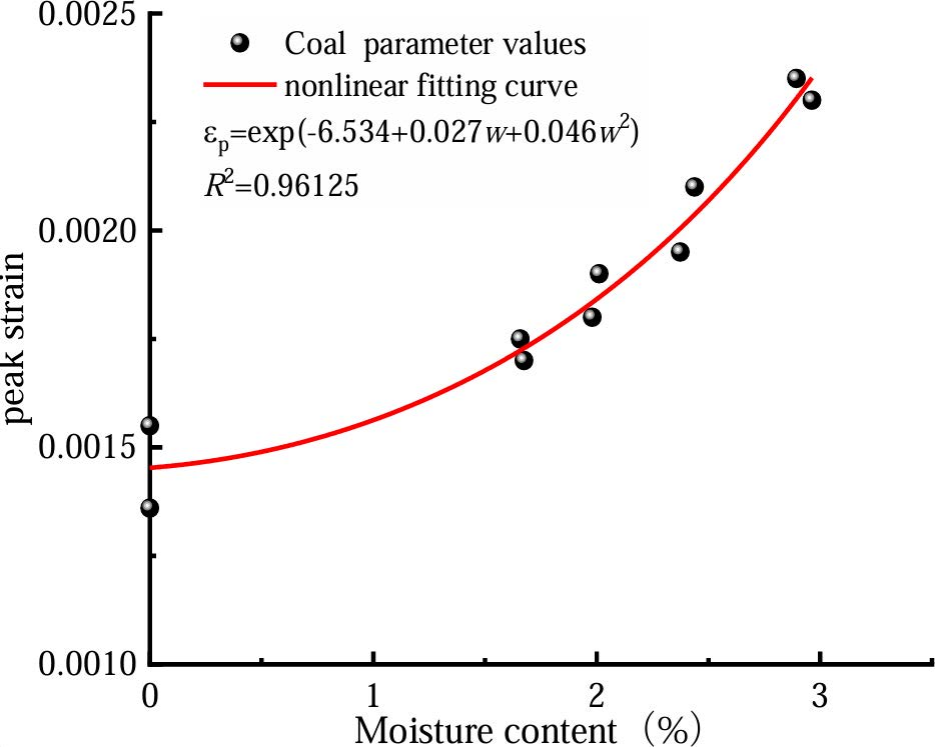
Trend of peak strain against water content in coal.

**Fig 7:**
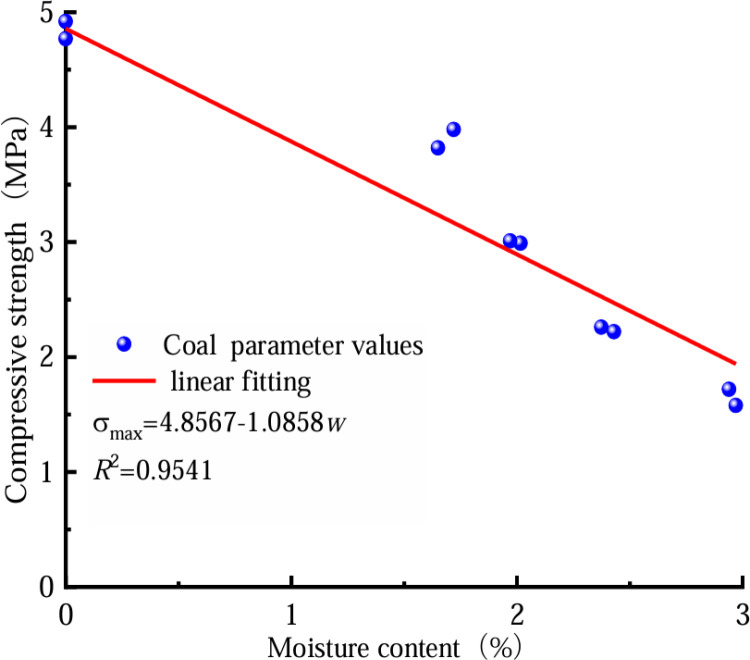
Trend of coal compressive strength against water content.

**Fig 8:**
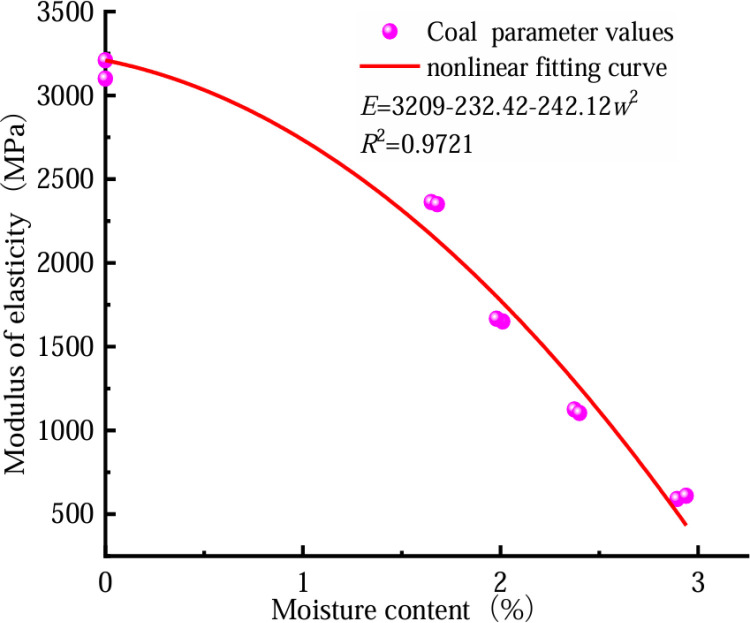
Trend of coal elastic modulus against water content.

In a bid to more effectively examine the coal’s reduced strength due to water absorption, we can analyze the coal’s stress–strain curve at varying moisture levels. This allows us to determine the three key mechanical properties: peak strain, compression modulus for the coal itself, and its corresponding elastic modulus when combined with rock at different hydration states. Subsequently, we can fit these three mechanical parameters. Figs 6–8 show the fitting relationship curves between the three mechanical parameters and the water content of the coal rock specimens.

Figs 5–7 demonstrate an inverse relationship between coal water content and both its compressive strength and elastic modulus. Conversely, a direct correlation is observed between the water content and the maximum coal strain. As the water content increases, the mechanical parameters decrease, from a rapid rate to a moderate rate. This result occurs because the micro-fractures of a coal body are filled with free water under highly watery conditions, and the free water flows fully with the full penetration of the crack tip, which considerably increases the coal’s transfer table from a state of brittleness to a state of plasticity. A significant relationship exists between coal’s moisture content and its mechanical characteristics and damage. The elastic modulus, compressive strength, peak strain, and water content of coal are expressed as follows.


$$\{\begin{array}{l} \varepsilon_{p}=\exp(a+bw+cw^{2})\\ \sigma =d+fw\\ E=h+i+jw^{2} \end{array}$$ (2)

where a,b,c,d,f,h,i,j are the peak strain, compressive strength, and modulus of elasticity of coal rocks with different water contents, which are the fitting parameters; *w* is the water content; *ε*_p_ is the peak strain; σ is the peak stress; *E* is the elastic modulus.

## 4 Energy evolution law of coal deformation and destruction

### 4.1 Energy calculation method

From an energy perspective, the deformation damage of a coal rock under external loading is a dynamic evolution process of energy input, accumulation, and dissipation, which is accompanied by energy dissipation during crack germination, expansion, and even macro-expansion of fissures [24]. Thus, examining the energy transformation patterns of coal with varying amounts of moisture content during its deformation and damage is crucial to uncovering the effect of water content on the damaged coal’s behavior. Fig 8 illustrates the evolution of the entire energy process throughout the coal destruction.

In the framework of thermodynamic laws, the energy resulting from work performed by an external force is expressed as follows [25]:


$$U_{0}=U_{d}+U_{\text{e}}$$ (3)

where $U_{0}$ is the total energy, $U_{\text{e}}$ is the elastic potential energy, and $U_{\text{d}}$ is the energy loss.

**Fig 9:**
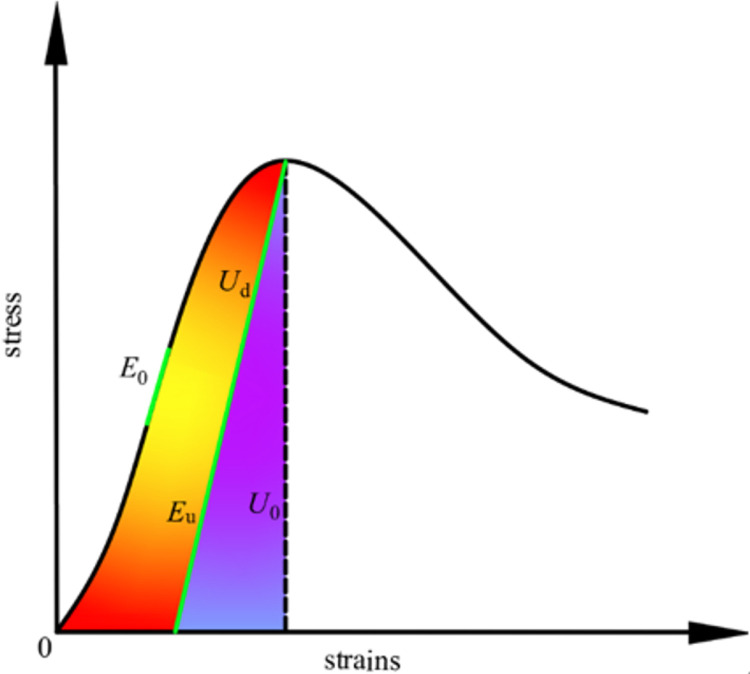
Evolutionary diagram of the entire energy process [26].


Fig 9 provided that there is no heat exchange between the system and its surroundings during the test, the energy derived from the external force $U_{0}$ is precisely the energy absorbed by the rock sample during uniaxial compression [27]:

$$\sigma_{2}=\sigma_{3}=0$$ (4)

$$U_{0}=\int_{0}^{\varepsilon_{1}}\sigma_{1}d\varepsilon_{1}+\int_{0}^{\varepsilon_{2}}\sigma_{2}d\varepsilon_{2}+\int_{0}^{\varepsilon_{3}}\sigma_{3}d\varepsilon_{3}=\int_{0}^{\varepsilon_{1}}\sigma_{1}d\varepsilon_{1}$$ (5)

$$U_{e}=\frac{1}{2}\sigma_{1}\varepsilon_{1}=\frac{\sigma_{1}^{2}}{2E_{u}}\approx \frac{\sigma_{1}^{2}}{2E_{0}}$$ (6)

where $\sigma_{1},\sigma_{2},\sigma_{3}$ are the maximum principal stress, intermediate principal stress, and minimum principal stress of the coal rock. $\varepsilon_{1},\varepsilon_{2}$, and $\varepsilon_{3}$ are the principal strains along the principal stresses. *E*u is the coal’s unloading modulus. $E_{0}$ is the actual modulus of elasticity of the coal rock.

### 4.2 Laws of energy evolution of coal rocks with different water contents

**Fig 10:**
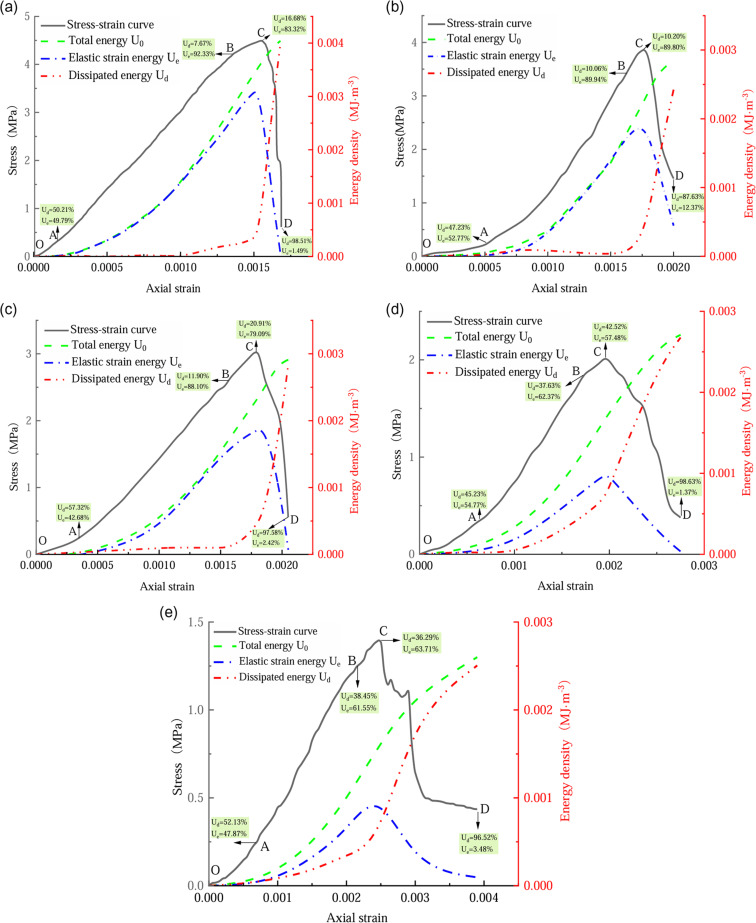
Energy evolution of damage processes in coal rocks with different water contents. (a) Energy evolution of coal rock with 0% water content. (b) Energy evolution of coal rock with 1.6575% water content. (c) Energy evolution diagram for coal rock with 2.021% water content. (d) Energy evolution of coal rock with 2.374% water content. (e) Energy evolution of coal rock with 2.972% water content.

Since only a small difference exists between the stress–strain curves of coal with similar water contents, we only examine one of the curves for each group in this paper. Based on the above energy calculation principle, Fig 10 presents the relationship between the energy transformation and stress–strain of coal with varying moisture levels during loading, deformation, and damage. The loading deformation damage process is divided into four stages according to the coal’s stress–strain relationship.

(1) Initial nonlinear stage (OA). No obvious difference is observed in the stress–strain curves at the initial loading stage of coal with different water contents, and the curve is concave. A gradual increase is observed in the overall energy density, along with the elastic strain energy and dissipation energy densities. This behavior is attributed to the presence of initial pores within the material, where the external load’s effort primarily facilitates the coalescence of these primary pores [28–30].
(2) Linear elastic stage (AB). The coal is close to the continuous medium after compacting, its linear elasticity characteristics are very obvious, and its total energy density and elastic strain energy density increase rapidly. Evidently, the energy absorbed from the surroundings is predominantly retained within the coal as elastic strain energy. Fig 8 illustrates that, under dry conditions, the rate of increase in the elastic strain energy in the AB segment of the coal is significant. The low, natural, and high water contents and water-saturated states are 2.67 × 10^‒3^MJ·m^‒3^·s^‒1^, 1.67 × 10^‒3^MJ·m^‒3^·s^‒1^, 1.618 × 10^‒3^MJ·m^‒3^·s^‒1^, 6.65 × 10^‒4^MJ·m^‒3^·s^‒1^, and 5.598 × 10^‒4^MJ·m^‒3^·s^‒1^, respectively. Therefore, in the linear elasticity stage, the ability of the coal to store elastic strain energy gradually decreases with increasing water content [31–32].
(3) Fissure development stage (BC). The nonlinear progression of the coal’s stress–strain curve is evident, and the specimen undergoes irreversible plastic deformation. The absorbed energy within the internal fissures and minute fracture expansions are primarily converted into dissipative forms. Consequently, a higher proportion of energy is dissipated while the proportion of the elastic strain energy diminishes, peaking at the point of maximum stress. Table 1 and 2 presents the characteristic values of energy at the peak value of coal with different water contents. The table shows that as the water content increases, the ultimate elastic strain energy of the coal at the peak stress decreases gradually, whereas the dissipation energy increases gradually. This behavior indicates that as the water content rises, coal undergoes an incrementally escalated extent of damage during its plastic phase.

**Table 1:** Raw coal I moisture content.

Specimen number	Moisture content/%
R_1–1_	0
R_1–2_	1.657
R_1–3_	2.021
R_1–4_	2.374
R_1–5_	2.972

**Table 2:** Raw coal II moisture content.

Specimen number	Moisture content/%
R_2–1_	0
R_2–2_	1.727
R_2–3_	1.980
R_2–4_	2.438
R_2–5_	2.963

(4) Post-peak damage stage (CD). Coal microcracks expand through the microfracture at point C into macrofracture, and the stress falls rapidly. The stored elastic strain energy is quickly discharged, considerably increasing the dissipated energy density. As this occurs, large cracks glide along the fracture surfaces, causing shear deformation. The entire strain energy assimilated throughout the coal’s destruction in dry conditions, a low water content, a natural water content, a high water content, and a water-saturated state is 4.04 × 10^‒3^MJ·m^‒3^, 2.87 × 10^‒3^MJ·m^‒3^, 2.84 × 10^‒3^MJ·m^‒3^, 2.71 × 10^‒3^MJ·m^‒3^, and 2.60 × 10^‒3^MJ·m^‒3^, respectively. These observations indicate that an increase in the moisture content of coal is correlated with a decrease in the amount of the total strain energy absorbed during the destruction process.

The development from coal damage to destruction is always accompanied by energy dissipation. Upon the fracture and enlargement of fissures in a coal rock, a portion of the accumulated elastic strain energy is transformed into dissipative energy. Table 3 shows that the ultimate elastic energy and dissipation energy of a dry coal rock peak at 3.07 × 10^‒3^ MJ·m^‒3^ and 3.50 × 10^‒4^ MJ·m^‒3^, respectively. Meanwhile, the ultimate elastic energy and dissipation energy of a coal rock in the saturated state peak at 9.37 × 10^‒4^MJ·m^‒3^ and 6.63 × 10^‒4^MJ·m^‒3^, respectively. Hence, an increased water content leads to a higher peak energy in a coal rock but results in reduced strain dissipation energy with lower elastic strain energy. Of the five groups of curves obtained, that of the dry coal rock exhibits the maximum total energy absorption, whereas the saturated coal rock has the minimum total absorbed energy. During actual mining, because of the rapid release of energy in the surrounding rock of a dry coal rock, geological disasters like rock ejection, collapse, and explosion can easily occur. Hence, the probability of rock explosion in the surrounding rock can be reduced by spraying high-pressure water.

**Table 3:** Peak energy of coal rock with different water contents.

Water content (%)	Ultimate elastic energy (MJ-m^‒3^)	Dissipated energy at peak (MJ-m^‒3^)	Total energy (MJ-m^‒3^)
0	3.07 × 10^‒3^	3.50 × 10^‒4^	4.04 × 10^‒3^
1.6575	2.12 × 10^‒3^	3.97 × 10^‒4^	2.87 × 10^‒3^
2.021	1.86 × 10^‒3^	4.75 × 10^‒4^	2.84 × 10^‒3^
2.374	1.10 × 10^‒3^	6.60 × 10^‒4^	2.71 × 10^‒3^
2.972	9.37 × 10^‒4^	6.63 × 10^‒4^	2.60 × 10^‒3^

## 5 Coal damage modeling based on the principle of minimum energy dissipation

The concept of minimal energy expenditure dictates that processes that require energy will always require the most efficient method within the bounds of their respective limitations. The term “corresponding constraints” pertains to the governing equations and criteria for resolution that must be fulfilled by the variables involved in the expression of the energy dissipation rate, ensuring that the energy dissipation rate is at a minimum [33].

If E,μ are set as the nominal modulus of elasticity and Poisson’s ratio, respectively, of the coal rock before the destructive energy dissipation occurs, then the ontological relationship before this occurrence is given as follows:


$$\{\begin{array}{c} \varepsilon_{1}=\frac{1}{E}[\sigma_{1}-\mu (\sigma_{2}+\sigma_{3})]\\ \varepsilon_{2}=\frac{1}{E}[\sigma_{2}-\mu (\sigma_{1}+\sigma_{3})]\\ \varepsilon_{3}=\frac{1}{E}[\sigma_{3}-\mu (\sigma_{1}+\sigma_{2})] \end{array}$$ (7)

The strain at any point throughout the process of energy dissipation caused by isotropic damage can be described by Equation (7), following the principle of strain equivalence [34].


$$\{\begin{array}{c} \varepsilon_{1}=\frac{1}{[1-D(t)]E}[\sigma_{1}-\mu (\sigma_{\text{2}}+\sigma_{3})]\\ \varepsilon_{2}=\frac{1}{[1-D(t)]E}[\sigma_{\text{2}}-\mu (\sigma_{1}+\sigma_{3})]\\ \varepsilon_{3}=\frac{1}{[1-D(t)]E}[\sigma_{3}-\mu (\sigma_{1}+\sigma_{2})] \end{array}$$ (8)

The energy dissipation rate at this point during the damage process due to $\sigma_{i}$ and caused by the damage variable D(t) can be expressed as


$$\varphi (t)=\sigma_{i}\dot{\varepsilon }\varepsilon_{i}^{N}(t)$$ (9)

where $\varepsilon_{i}^{N}(t)$ is the irreversible strain rate caused by D(t); *t* is the time parameter indicating the damage destruction process. From the above equation, we have


$$\{\begin{array}{c} \varepsilon_{1}=\frac{-\dot{D}(t)}{[1-D(t)]E}[\sigma_{1}-\mu (\sigma_{2}+\sigma_{3})]\\ \varepsilon_{2}=\frac{-\dot{D}(t)}{[1-D(t)]E}[\sigma_{2}-\mu (\sigma_{1}+\sigma_{3})]\\ \varepsilon_{3}=\frac{-\dot{D}(t)}{[1-D(t)]E}[\sigma_{3}-\mu (\sigma_{1}+\sigma_{2})] \end{array}$$ (10)

Substituting Equation (10) into Equation (9) yields


$$\varphi (t)=-\frac{\dot{D}(t)}{{\left[1-D(t)\right]}^{2}E}[\sigma_{1}^{2}+\sigma_{2}^{2}+\sigma_{3}^{2}-2\mu (\sigma_{1}\sigma_{2}+\sigma_{2}\sigma_{3}+\sigma_{3}\sigma_{1})]$$ (11)

It is known that under the uniaxial compression of a coal rock,


$$\sigma_{1}=\sigma$$ (12)

$$\sigma_{2}=\sigma_{3}=0$$ (13)

The energy consumption rate is


$$\varphi (t)=-\frac{\dot{D}(t)}{{\left[1-D(t)\right]}^{2}E}\sigma^{2}$$ (14)

The limitations experienced by a coal rock during the energy consumption process can be summarized in the following manner:


$$F(\sigma )=\sigma -R_{c}=0$$ (15)

where $R_{c}$ is the uniaxial compressive strength of the coal rock.

In line with the principle of minimal energy usage, Equation (14) should satisfy the restriction of taking the stationary value under the condition of Equation (15), which can be obtained as follows:


$$\frac{\partial [\varphi (t)+\lambda F]}{\partial \sigma }=0$$ (16)

According to the mechanics of continuum damage, it is known that [35]


σ=E(1−D)ε (17)

From Equation (14) to Equation (17), we obtain


$$D(t)=1-e^{\left(\frac{\lambda }{2\varepsilon }+c\right)}$$ (18)

From Fig 8, the following geometrical relationship can be observed:


$$\{\begin{array}{l} \varepsilon =\varepsilon_{P},\sigma =\sigma_{\max}\\ \varepsilon =\varepsilon_{P},\frac{d\sigma }{d\varepsilon }=0 \end{array}$$ (19)

**Fig 11:**
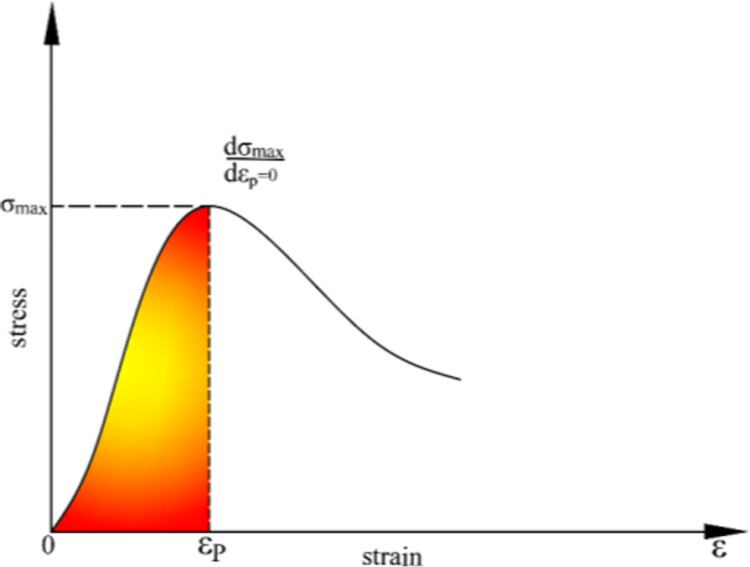
Simple illustration diagram.


In Fig 11, $\varepsilon_{P}$ is the peak strain value; $\sigma_{\max}$ is the peak stress value, which can be calculated and obtained as follows:

$$\lambda =2\varepsilon_{p}$$ (20)

$$c=\ln\sigma_{\max}-\ln Ee\varepsilon p$$ (21)

Substituting Equation (20) and Equation (21) into Equation (18) yields the coal rock damage evolution model:


$$D(t)=1-e^{\left(\frac{\varepsilon_{p}}{\varepsilon }+\ln\sigma_{\max}-\ln Ee\varepsilon_{p}\right)}$$ (22)

Substituting Equation (2) into the above equation, we obtain the damage evolution model of coal rock under different water content rates:


$$D(t)=1-\exp\{\frac{\exp(a+bw+cw^{2})}{\varepsilon }+\ln[(d+fw)-\ln(h+i+fw^{2})e\exp(a+bw+cw^{2})]\}$$ (23)

### 5.1 Determination of initial value of damage and critical value of damage

When a coal rock undergoes no damage,


$$D=1-\exp(\frac{\lambda }{2\varepsilon }+c)=0$$ (24)

That is, the initial value of damage to a coal rock with different water contents is


$$\varepsilon_{0}=-\frac{\lambda }{2c}=\frac{2\varepsilon_{p}}{2(\ln Ee\varepsilon_{p}-\ln\sigma_{m})}=\frac{\exp(a+bw+cw^{2})}{\ln[(h+i+jw^{2})\text{e}\exp(a+bw+cw^{2})]-\ln(d+fw)}$$ (25)

Apparently,


$$\varepsilon_{0}<\varepsilon_{p}$$ (26)

It can be seen that damage already exists in the coal rock before it reaches the destruction stage. On the stress–strain diagram, a critical juncture separates elasticity and plasticity. The threshold value of damage marks the distinction between elastic and plastic damage to coal rock materials.

When ε→∞, we combine Equation (24) with Equation (2) to obtain the damage critical value of coal rocks with different water contents.


$$D=1-\exp\{\ln(d+fw)-\ln[(h+i+jw^{2})\text{e}\exp(a+bw+cw^{2})]\}$$ (27)

**Fig 12:**
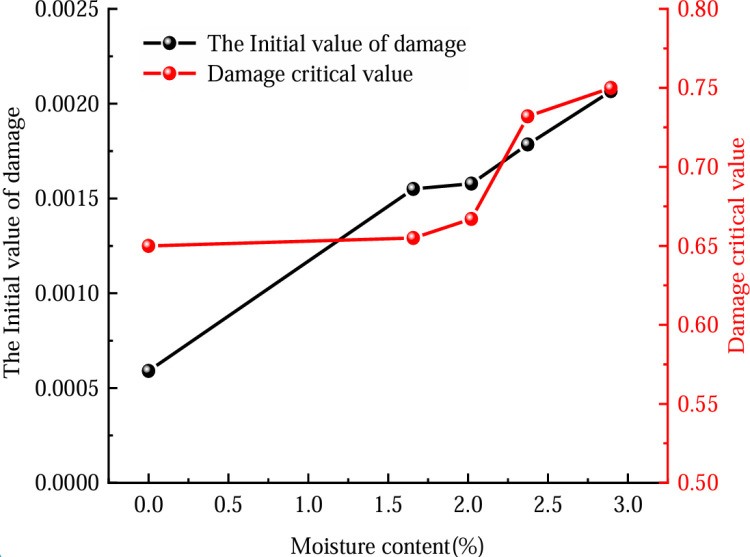
Evolution law of initial damage value and critical damage value of coal under different water contents.

The fitting parameters *a* = ‒6.534, *b* = 0.27, *c* = 0.046, *d* = 4.8567, *f* = ‒1.0858, *h* = 3209, *i* = ‒232.442, j = ‒242.1 obtained from the experiments are substituted into Equation (25) and Equation (27) to obtain the initial value of damage and damage critical value for coal with different water contents, as shown in Fig 12.

If a coal rock enters its destruction phase, its capacity to bear loads is hypothesized to diminish entirely. Consequently, the practical load-bearing surface area should be reduced to zero. Although the damage parameter pertains to the coal rock, it retains a degree of load-bearing capability post-destruction. This signifies the existence of residual strength, and its active load-bearing zone should exceed zero in magnitude. Thus, the critical threshold for damage must be under 1. Equation (24) reveals that both the initial and critical damage values are solely influenced by the peak strain, compressive strength, and elastic modulus, serving as an aggregated indicator of these properties. Fig 12 illustrates a positive correlation between coal moisture content and coal initiation and critical damage levels, with the relationship being non-linear.

### 5.2 Stress–strain principal model of coal based on the principle of minimum energy dissipation

The results reported by Lu Zu De [36] show that the stress–strain relationship in the compaction stage of a coal rock can be expressed as


$$\sigma =\sigma_{A}{\left(\varepsilon /\varepsilon_{A}\right)}^{2}$$ (28)

where σ,ε are the stress and strain in uniaxial compression, respectively; $\sigma_{A},\varepsilon_{A}$ are the maximum stress and strain in the compression stage, respectively.

Substituting Equation (22) into Equation (17) yields


$$\sigma =E\varepsilon \text{exp(}\frac{\varepsilon_{p}}{\varepsilon }+\ln\sigma_{m}-\ln Ee\varepsilon_{p})$$ (29)

**Fig 13:**
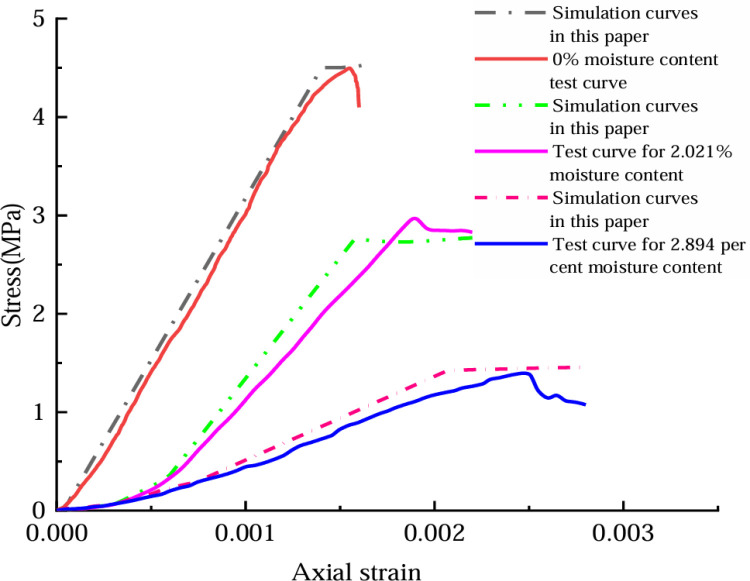
Simulation curve and uniaxial compression test curve.

**Fig 14:**
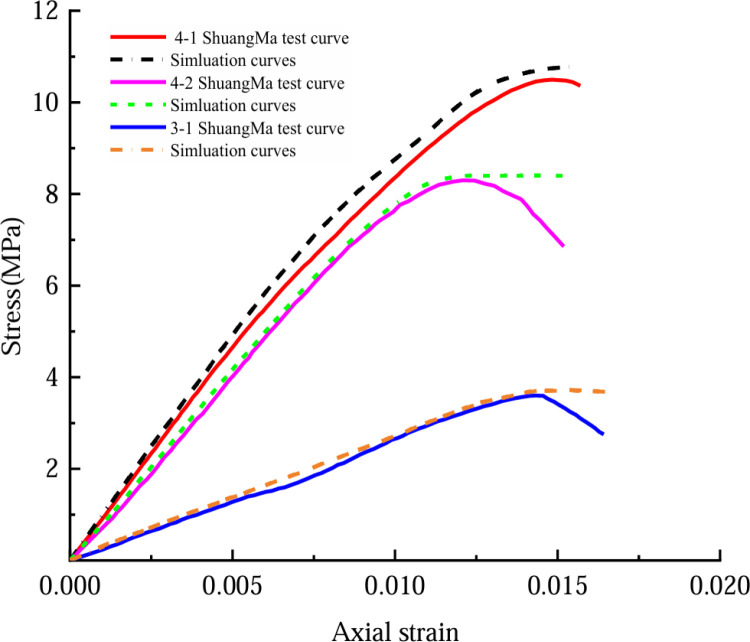
Simulation curve and Shuangma No. 2 mine test curve comparison.


In this paper, some uniaxial compression test curves are used to validate the derived ontology modeling. Figs 13–14 show the simulation curves and test curves for 0%, 2.021%, and 2.894% moisture contents of coal rock. The fitted parameters *a* = ‒6.534, *b* = 0.27, *c* = 0.046, *f* = ‒1.0858, *h* = 3209, *i* = ‒232.442, *j* = ‒232.1, and *g* =  ‒232.42; the uniaxial compression test data were analyzed to determine the peak stress and strain values during the compaction phase for the coal rock samples with varying moisture contents. By inserting the specified parameters into Equation (29), we derive the segmented intrinsic stress–strain model of coal rock from uniaxial compression tests at varying water contents.

$$\{\begin{array}{l} \sigma =\sigma_{A}{\left(\varepsilon /\varepsilon_{A}\right)}^{2}\;\varepsilon \leq \varepsilon_{A}\\ \sigma =(d+fw)\varepsilon \;\varepsilon_{A}<\varepsilon <\varepsilon_{0}\\ \text{σ}=\text{ε}(h+i+jw^{2})\exp[\frac{\exp(a+bw+cw^{2})}{\text{ε}}+\ln(d+fw)-\ln(h+i+jw^{2})e\exp(a+bw+cw^{2})]\varepsilon \geq \varepsilon_{0} \end{array}$$ (30)

The conventional developmental model, rooted in the principle of minimal energy use, overlooks the compression phase; hence, when a coal rock remains in the initial compression stage for an extended period, a considerable deviation occurs. This deviation results in a misalignment between the theoretical curve and the actual uniaxial test stress–strain curve. Consequently, this model is more fitting for simulating segmented coal damage development that includes an elongated elasticity period. In contrast, the coal rock damage development model based on minimum energy expenditure, as investigated in this study, is more suitable for analyzing the uniaxial compression’s stress–strain dynamics under varied moisture levels. This study introduces a coal damage ontology model based on the principle of minimum energy dissipation, proving more suitable for examining the uniaxial compressive stress–strain behavior of coal in various water content scenarios.

## 6 A comparative study of the ontology model

**Fig 15:**
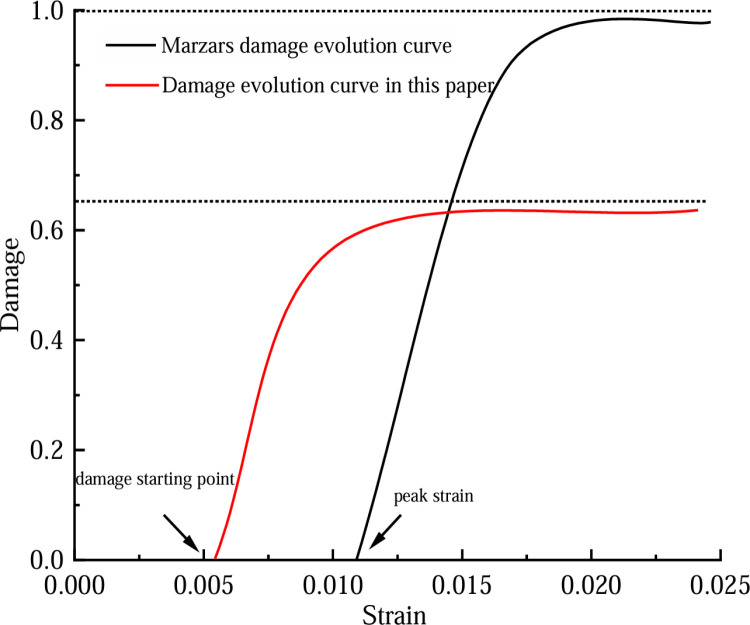
Comparison of evolution law of damage variable.

The traditional Marzars damage model and the novel damage model introduced in this paper can be utilized to examine the detailed constitutive behavior of coal rock damage through stress–strain relationships derived from rock testing. As such, both models share strong parallels [37]. According to the Marzars damage model, the stress–strain curve for rock-like materials is segmented into two distinct parts. The section leading up to the peak stress is assumed to exhibit a linear relationship. If the strain ε_p_ corresponding to the peak stress σ_max_ is the strain at the beginning of the damage, then, when ε≤ε_p_, the Marzars damage model considers that there is no damage at this time, that is, D = 0. When ε>ε_p_, the Marzars damage model considers that the strain decays exponentially at this time, and damage occurs inside the rock material, that is, D > 0. This stage is also the stage of macroscopic crack formation and rapid instability failure in a rock. In reality, the coal rock mass already experiences internal crack initiation due to inherent flaws before reaching its ultimate stress limit. At this juncture, damage progression is underway. Therefore, the damage threshold should be before the peak stress, not at the peak stress. The Marzars model holds that the critical value of rock damage D =  1, that is, the effective bearing area of rock material is 0, while the critical value of model damage D proposed in this paper is less than 1. Obviously, the conclusion of this paper is more in line with the actual situation of coal failure, because in practice, the coal body still has residual strength after failure and can still bear. It can be seen that the effective bearing capacity is not equal to 0 at this time, as shown in Fig 15.

## 7 Conclusion and foresight

(1) From an energy perspective, a rise in the moisture content of coal is accompanied by a gradual reduction in its capacity to store strain energy elastically. The fraction of dissipated energy within the total strain energy of coal increases, and the cumulative strain energy absorbed by the coal rock during its failure process declines, suggesting a diminishing intensity of coal’s destructiveness.
(2) The principle of minimum dissipated energy was used to establish a damage ontology model of coal rocks with different water contents. When $\varepsilon_{0}<\varepsilon_{p}$, the damage of coal rock D>0, indicating that the damage only starts to develop before the peak value. When ε→∞, the onset and critical points of damage are solely linked to the material’s compressive strength, peak strain, and elastic modulus, serving as a composite indicator encompassing all three attributes. The initial and critical damage values of a coal rock exhibit a positive nonlinear association with its moisture levels.
(3) Utilizing the principle of minimum energy expenditure, we developed an ontology-based stress–strain model for coal rocks with varying levels of water content under one-dimensional compression. This approach is an improvement upon conventional models as it addresses their inaccuracies in simulating materials that spend a lengthy period in the compaction stage. The model can clearly express the elastic–plastic cut-off point of coal rocks with different water contents.
(4) The energy evolution mechanism of coal under different water contents was studied, which is the internal mechanism of preventing rock bursts by water injection in underground coal mining. It also provides a certain reference value for the prediction and evaluation of water disaster and the rock damage assessment of coal and rock pillars.
(5) Investigating the dynamics of energy and damage transformations in coal with varying moisture levels is pivotal for understanding the fundamental process behind utilizing rock water injection as a preventive measure against rock bursts in underground coal mines. It also provides a reference value for the prediction and evaluation of water damage in coal rock pillars, the secondary development of FLAC3D and PFC3D numerical simulation software, and the evaluation of rock damage.
